# Supplementary material for: Incidence of SARS-CoV-2 infection among healthcare workers before and after COVID-19 vaccination in a tertiary paediatric hospital in Warsaw: A retrospective cohort study
Source: PLoS One. 2024 May 23;19(5):e0301612. doi: 10.1371/journal.pone.0301612 (PMC11115228; doi:10.1371/journal.pone.0301612)
Supplement: S1 File — (DOCX) [file pone.0301612.s001.docx]

Supporting material

**Supplementary Methods:**

| STUDY DESIGN:  **Conceptual proposition:** to identify factors associated with HCW SARS-CoV-2 infection which make it possible to direct the management of health care services.  **Research objective:** to analyse the incidence of new SARS-CoV-2 infection among HCWs (before and after vaccination with BNT162b2) and to explore demographic and occupational factors associated with SARS-CoV-2 infection. To analyse the vaccine effectiveness against any SARS-CoV-2 infection in HCWs.  **Research definition:** a cohort, retrospective study with secondary data, conducted with the Strengthening the Reporting of Observational Studies in Epidemiology (STROBE) guidelines for reporting cohort studies^[[1]](#footnote-1)^.  **Setting:** a tertiary paediatric hospital in Warsaw.  **Participants:** all HCWs (including clinical and non-clinical) with at least one PCR test performed as part of universal screening were included.  VARIABLES AND SAMPLES:  **Observed variables:** age, gender, profession, workplace, vaccination status, voluntary SARS-CoV-2 nucleocapsid antibody test results (performed before implementation of the HCW vaccination programme in January 2021), SARS-CoV-2 RNA results from longitudinal universal screening (between October 20, 2020, and May 6, 2021, which correspond to epidemiological (epi) week 43, 2020 and epi week 18, 2021, a period covering the second and third epidemic waves in Poland), and SARS-CoV-2 RNA results from contact tracing, symptomatic testing, or testing upon return to work after sick leave (due to respiratory illness) collected from October 20, 2020, up to August 31, 2021 (epi week 43, 2020 to epi week 35, 2021).  **Data source:** prospectively maintained laboratory records, the HR database, the ICP records (including routinely collected epidemiological data on laboratory confirmed SARS-CoV-2 infections among HCWs).  DATA ANALYSIS:  **Preliminary analysis:** median with interquartile range (IQR) of continuous variables and proportions of dichotomous variables.  **Statistical procedures:** incidence of SARS-CoV-2 infections in HCWs calculated by demographic parameters, profession, workplace, and vaccination status.  Factors associated with SARS-CoV-2 infection were examined using logistic regression models. |
| --- |

**Study population and definition of follow-up period for the incidence of SARS-CoV-2 infection in HCWs before vaccination (study phase 1)**

Follow-up began with the beginning of the universal screening programme on October 20, 2020, and continued until receipt of the first vaccine dose or on February 28, 2021 (whichever occurred earlier).

Both the results of the SARS-CoV-2 RT-PCR test performed as part of the universal screening programme and the tests performed outside of the screening were included in the analysis. In addition, positive results of the tests performed outside the CMHI during the study period, extracted from the database maintained by the ICP team, were also included.

To calculate the incidence of new SARS-CoV-2 infection, HCWs with previous SARS-CoV-2 infection (based on positive PCR test results and/or positive IgG anti-nucleocapsid protein) or with continuous positive RT-PCR results were excluded from the incidence analysis.

**Study population and definition of the follow-up period for the incidence of SARS-CoV-2 infection in HCWs after implementation of a vaccination programme (study phase 2)**

The HCW vaccination programme began on January 4, 2021, starting with the Pfizer-BioNtech BNT162b2 vaccine. In this study, one type of the SARS-CoV-2 vaccine was considered. For incidence analysis, we used a wash-out period of 0–13 days after each dose of vaccine and adopted the ECDC’s criteria to determine vaccination status at the time of PCR testing^[[2]](#footnote-2)^. The HCWs were classified as fully vaccinated (≥14 days after dose 2), partially vaccinated (≥14 days after dose 1 and < 14 days after dose 2) or unvaccinated (no vaccine dose or < 14 days after dose 1).

Incidence analysis was performed for HCWs with known vaccination status and separately in subgroups of unvaccinated, partially, and fully vaccinated HCWs. The follow-up period for fully vaccinated HCWs ended on August 31, 2021, or at the time of the first PCR positive test result, whichever occurred earlier. The follow-up period for partially vaccinated HCWs ended on August 31, 2021, or at the time of the first PCR positive test result, or at the receipt of vaccine dose 2, whichever occurred earlier. The follow-up period for unvaccinated HCWs was from March 1, 2021, to the first PCR positive test result, at the receipt of vaccine dose 1, or on August 31, 2021, whichever occurred earlier.

In study phase 2, PCR testing was mostly symptom based, and the available data and number of events do not support VE stratification by symptom or epi week.

# Supplementary Tables

**Table S1** Characteristics of the HCWs by SARS-CoV-2 infection status including 86 previously infected HCWs (sensitivity analysis, n = 2233)

| Characteristics | Infected | Uninfected | p-value |
| --- | --- | --- | --- |
| Total, n (%): | 443 (19.8) | 1790 (70.2) |  |
| Age, median (IQR), years: | 47.0 (37.8–54.7) | 47.3 (36.6–55.8) | 0.651 |
| Female gender, n (%): | 385 (20) | 1 495 (80.0) | 0.080 |
| Professional category, n (%): |  |  | < 0.001 |
| nurse | 176 (24.0) | 556 (76.0) |  |
| physician | 64 (13.0) | 413 (87.0) |  |
| other with direct patient contact | 62 (21.0) | 234 (79.0) |  |
| other without direct patient contact | 141 (19.0) | 587 (81.0) |  |
| Working in COVID-19 area, n (%): |  |  | 0.058 |
| yes | 45 (25.0) | 133 (75.0) |  |
| no | 398 (19.0) | 1657 (81.0) |  |
| Hospital department, n (%): |  |  | 0.569 |
| clinical | 325 (20.0) | 1289 (80.0) |  |
| non-clinical | 118(19.0) | 501 (81.0) |  |
| Wards, n (%): |  |  | 0.069 |
| medical | 187 (20.0) | 752 (80.0) |  |
| surgical | 41 (22.0) | 142 (78.0) |  |
| intensive care | 33 (27.0) | 89 (73.0) |  |
| auxiliary | 38 (15.0) | 208 (85.0) |  |
| ambulatory | 26 (21.0) | 98 (79.0) |  |
| laboratory | 28 (22.0) | 99 (78.0) |  |
| maintenance | 26 (27.0) | 71 (73.0) |  |
| administration | 55 (16.0) | 288 (84.0) |  |
| other | 9 (17.0) | 43 (83.0) |  |
| Symptoms, n (%): |  |  |  |
| yes | 190 (42.9) | - |  |
| no | 231 (52.1) | - |  |
| unknown | 22 (4.9) | - |  |

Abbreviation: IQR – interquartile range

The percentages were presented in rows to highlight the proportion of infected and uninfected HCWs for each level of the variables (not applicable to the symptoms category)

**Table S2** Association of demographic and occupational characteristics of the HCWs with SARS-CoV-2 infection before vaccination (study phase 1)

| Characteristics | Total  n = 2147 | Infected, n (%)  n = 357 (16.6) | OR (95% CI) | p-value | aOR (95% CI) ^a,b^ | p-value |
| --- | --- | --- | --- | --- | --- | --- |
| Gender: |  |  |  |  |  |  |
| male | 341 | 46 (13.5) | ref |  |  |  |
| female | 1806 | 311 (17.2) | 1.33 (0.96–1.86) | 0.091 | 1.08 (0.72–1.62) | 0.703 |
| Median age (IQR), years: | 47.2 (36.6–55.6) | 46.6 (37.1–54.8) | 1.00 (0.99–1.01) | 0.532 |  |  |
| Professional category: |  |  |  |  |  |  |
| nurse | 695 | 139 (20.0) | 1.22 (0.93–1.60) | 0.145 | 1.80 (1.29–2.52) | 0.001 |
| physician | 459 | 46 (10.0) | 0.55 (0.38–0.78) | 0.001 | 0.45 (0.30–0.68) | < 0.001 |
| other with direct patient contact | 286 | 52 (18.2) | 1.09 (0.76–1.56) | 0.649 | 0.78 (0.51–1.20) | 0.260 |
| other without direct patient contact | 707 | 120 (17.0) | ref |  |  |  |
| Hospital department: |  |  |  |  |  |  |
| clinical | 1549 | 260 (16.8) | 1.04 (0.81–1.34) | 0.753 | - | - |
| non-clinical | 598 | 97 (16.2) | ref |  |  |  |
| Working in COVID-19 area: |  |  |  |  |  |  |
| yes | 165 | 32 (19.4) | 1.23 (0.82–1.84) | 0.321 | - | - |
| no | 1982 | 325 (16.4) | ref |  |  |  |
| Wards: |  |  |  |  |  |  |
| medical | 901 | 149 (16.5) | 1.17 (0.82–1.65) | 0.394 | - | - |
| surgical | 172 | 30 (17.4) | 1.24 (0.76–2.04) | 0.393 | - | - |
| intensive care | 111 | 22 (19.8) | 1.45 (0.83–2.53) | 0.188 | - | - |
| auxiliary | 242 | 34 (14.1) | 0.96 (0.60–1.54) | 0.868 | - | - |
| ambulatory | 123 | 25 (20.3) | 1.50 (0.88–2.56) | 0.137 | - | - |
| laboratory | 120 | 21 (17.5) | 1.25 (0.71–2.18) | 0.440 | - | - |
| maintenance | 90 | 19 (21.1) | 1.57 (0.87–2.84) | 0.132 | - | - |
| administration | 337 | 49 (14.5) | Ref |  |  |  |
| other | 51 | 8 (15.7) | 1.09 (0.49–2.47) | 0.829 | - | - |
| Median no. of PCR tests per person, n (IQR) | 4 (3–5) | 2 (1–3) | 0.37 (0.34–0.41) | < 0.001 | 0.35 (0.31–0.39) | < 0.001 |

Abbreviations: OR – odds ratio; aOR – adjusted odds ratio; CI – confidence interval; IQR – interquartile range; ref – reference category

^a^ adjustment for age, gender, and number of PCR tests per person

^b^ “-”, variable not included in the multivariate analysis model

**Table S3** Characteristics of vaccinated and unvaccinated HCWs (n = 1461)

| Characteristics | Vaccinated ^a^ | Unvaccinated ^b^ | p-value |
| --- | --- | --- | --- |
| Total, n (%) | 1345 | 116 |  |
| Age, median (IQR), years: | 48.6 (38.2–57.0) | 46.8 (35.8–54.3) | 0.001 |
| Female gender, n (%) | 1128 (91.8) | 101 (8.2) | 0.365 |
| Professional category, n (%): |  |  | < 0.001 |
| nurse | 407 (92.5) | 33 (7.5) |  |
| physician | 327 (97.6) | 8 (2.4) |  |
| other with direct patient contact | 163(89.1) | 20 (10.9) |  |
| other without direct patient contact | 448 (89.1) | 55 (10.9) |  |
| Working in COVID-19 area, n (%): |  |  | 0.804 |
| yes | 96 (91.4) | 9 (8.6) |  |
| no | 1249 (92.1) | 107 (7.9) |  |
| Hospital department, n (%): |  |  | 0.006 |
| clinical | 963 (93.3) | 69(6.7) |  |
| non-clinical | 382 (89.0) | 47(11.0) |  |
| Wards, n (%): |  |  | 0.224 |
| medical | 569 (93.0) | 43 (7.0) |  |
| surgical | 113 (96.6) | 4 (3.4) |  |
| intensive care | 62 (92.5) | 5 (7.5) |  |
| auxiliary | 147 (93.6) | 10 (6.4) |  |
| ambulatory | 73 (91.3) | 7 (8.7) |  |
| laboratory | 86 (90.5) | 9 (9.5) |  |
| maintenance | 53 (86.9) | 8 (13.1) |  |
| administration | 213 (89.1) | 26 (10.9) |  |
| other | 30 (88.2) | 4 (11.8) |  |

Abbreviation: OR – odds ratio.

^a^ received at least one dose of the BNT162b2 vaccine between January 4, 2021, and February 28, 2021

^b^ did not receive any dose of vaccine between January 4, 2021, and February 28, 2021

***NOTE*:** the definition of vaccination status was different for the incidence study in which the status at the time of PCR testing was considered

**Table S4** Characteristics of the HCWs stratified by SARS-CoV-2 infection status during study phase 2 (postvaccination; n = 1461)

| Characteristics | Infected | Uninfected | Total | p-value |
| --- | --- | --- | --- | --- |
| Total, n (%) | 93 (6.4) | 1368 (93.6) | 1461 |  |
| Age, median (IQR), years: | 45.1 (35.9–53.3) | 48.6 (38.1–56.9) | 48.3 (37.8–56.6) | 0.062 |
| Female gender, n (%): | 81 (6.6) | 1 148 (93.4) | 1 229 | 0.417 |
| Professional category, n (%): |  |  |  | 0.544 |
| nurse | 30 (6.8) | 410 (93.2) | 440 |  |
| physician | 16 (4.8) | 319 (95.2) | 335 |  |
| other with direct patient contact | 11 (6.0) | 172 (94.0) | 183 |  |
| other without direct patient contact | 36 (7.2) | 467 (92.8) | 503 |  |
| Hospital department, n (%): |  |  |  | 0.690 |
| clinical | 64 (6.2) | 968 (93.8) | 1032 |  |
| non-clinical | 29 (6.8) | 400 (93.2) | 429 |  |
| Working in COVID-19 area, n (%): |  |  |  | 0.485 |
| yes | 5 (4.8) | 100 (95.2) | 105 |  |
| no | 88 (6.5) | 1268 (93.5) | 1356 |  |
| Wards, n (%): |  |  |  | 0.771 |
| medical | 38 (6.2) | 573 (93.8) | 611 |  |
| surgical | 6 (5.1) | 111 (94.9) | 117 |  |
| intensive care | 4 (6.0) | 63 (94.0) | 67 |  |
| auxiliary | 9 (5.7) | 148 (94.3) | 157 |  |
| ambulatory | 7 (8.8) | 73 (91.2) | 80 |  |
| laboratory | 4 (4.2) | 91 (95.8) | 95 |  |
| maintenance | 2 (3.3) | 59 (96.7) | 61 |  |
| administration | 20 (8.4) | 219 (91.6) | 239 |  |
| other | 3 (8.8) | 31 (91.2) | 34 |  |
| Vaccination status, n (%): |  |  |  | < 0.001 |
| unvaccinated | 50 (39.7) | 76 (60.3) | 126 |  |
| partially vaccinated | 6 (12.2) | 43 (87.8) | 49 |  |
| fully vaccinated | 37 (2.9) | 1 249 (97.1) | 1286 |  |
| Symptoms at positive PCR test result, n (%): |  |  |  |  |
| total | 69 (75.0) | - | 69 |  |
| unvaccinated | 38 (76.0) | - | 38 | 0.864 |
| partially vaccinated | 4 (66.7) | - | 4 |  |
| fully vaccinated | 27 (73.0) | - | 27 |  |
| Median no. of PCR tests per person, n (IQR) | 2 (1–3) | 2 (2–3) | 2 (2–3) | 0.002 |

Abbreviation: OR – odds ratio

**Table S5** Association of vaccination status with primary laboratory confirmed SARS-CoV-2 infection, after inclusion of additional category of HCWs with missing vaccination status

|  | Total,  n | Infected,  n (%) | Uninfected,  n (%) | p-value |
| --- | --- | --- | --- | --- |
| Vaccination status: |  |  |  | < 0.001 |
| fully vaccinated | 1286 | 37 (2.9) | 1249 (97.1) |  |
| partially vaccinated | 49 | 6 (12.2) | 43 (87.8) |  |
| unvaccinated | 126 | 50 (39.7) | 76 (60.3) |  |
| unknown | 192 | 0 (0) | 192 (100) |  |

**Table S6** Association of demographic and occupational characteristics of HCWs with SARS-CoV-2 infection after vaccination (study phase 2)

| Characteristics | Total, n = 1461 | Infected, n = 93 | OR (95% CI) | P-value | aOR (95% CI)^a, b^ | p-value |
| --- | --- | --- | --- | --- | --- | --- |
| Median age (IQR), years | 48.3 (37.8–56.6) | 45.1 (35.9–53.3) | 0.98 (0.96–1.00) | 0.016 | 0.99 (0.97–1.01) | 0.201 |
| Gender, n (%): |  |  |  |  |  |  |
| male | 231 | 12 (5.2) | ref |  |  |  |
| female | 1229 | 81 (6.6) | 1.29 (0.69–2.41) | 0.418 | 1.15 (0.58–2.28) | 0.693 |
| Professional category, n (%): |  |  |  |  |  |  |
| nurse | 440 | 30 (6.8) | 0.95 (0.57–1.57) | 0.839 | - | - |
| physician | 335 | 16 (4.8) | 0.65 (0.36–1.19) | 0.164 | - | - |
| other with direct patient contact | 183 | 11 (6.0) | 0.83 (0.41–1.67) | 0.580 | - | - |
| other without direct patient contact | 503 | 36 (7.2) | ref |  |  |  |
| Hospital department, n (%): |  |  |  |  |  |  |
| clinical | 1032 | 64 (6.2) | 0.91 (0.58–1.44) | 0.691 | - | - |
| non-clinical | 429 | 29 (6.8) | ref |  |  |  |
| Working in COVID-19 area, n (%): |  |  |  |  |  |  |
| yes | 105 | 5 (4.8) | 0.72 (0.29–1.82) | 0.487 | - | - |
| no | 1356 | 88 (6.5) | ref |  |  |  |
| Wards, n (%): |  |  |  |  | - | - |
| medical | 611 | 38 (6.2) | 0.73 (0.41–1.28) | 0.266 | - | - |
| surgical | 117 | 6 (5.1) | 0.59 (0.23–1.52) | 0.274 | - | - |
| intensive care | 67 | 4 (6.0) | 0.70 (0.23–2.11) | 0.521 | - | - |
| auxiliary | 157 | 9 (5.7) | 0.67 (0.30–1.50) | 0.327 | - | - |
| ambulatory | 80 | 7 (8.8) | 1.05 (0.43–2.58) | 0.915 | - | - |
| laboratory | 95 | 4 (4.2) | 0.48 (0.16–1.45) | 0.193 | - | - |
| maintenance | 61 | 2 (3.3) | 0.37 (0.08–1.63) | 0.190 | - | - |
| administration | 239 | 20 (8.4) | ref |  |  |  |
| other | 34 | 3 (8.8) | 1.06 (0.30–3.78) | 0.929 | - | - |
| Vaccination status, n (%): |  |  |  |  |  |  |
| unvaccinated | 126 | 50 (39.7) | ref |  | ref |  |
| partially vaccinated | 49 | 6 (12.2) | 0.21 (0.08–0.54) | 0.001 | 0.21 (0.08–0.54) | 0.001 |
| fully vaccinated | 1286 | 37 (2.9) | 0.05 (0.03–0.07) | < 0.001 | 0.05 (0.03–0.09) | < 0.001 |
| Median no. of PCR tests per person, n (IQR) | 2 (2–3) | 2 (1–3) | 0.48 (0.37–0.62) | < 0.001 | 0.93 (0.70–1.24) | 0.628 |

Abbreviations: OR – odds ratio; aOR – adjusted odds ratio; CI – confidence interval; ref – reference category

^a^ adjustment for age, gender, and number of PCR tests per person

^b^ “-”, variable not included in the multivariate analysis model

## Supplementary Figures

HCW SARS-CoV-2 PCR testing


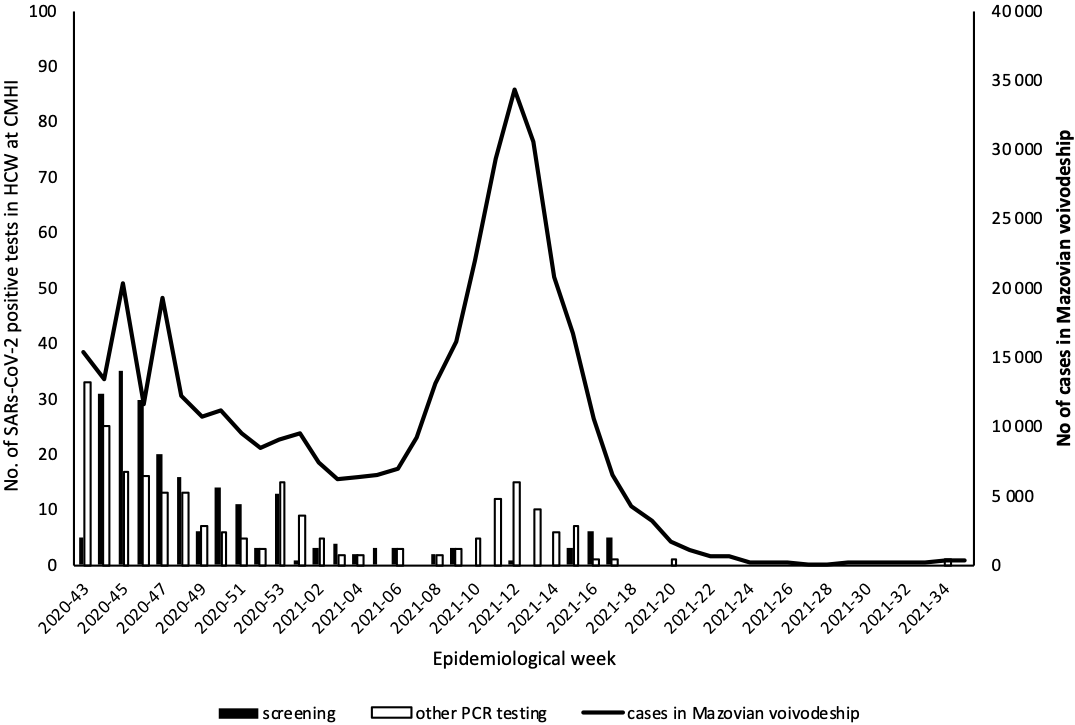


HCW vaccination programme at CMHI

Universal HCW screening (week 43, 2020 – week 18, 2021)

Dominant Delta variant (B.1.617.2)

**Figure S1** New weekly cases of SARS-CoV-2 infections by testing mode among HCWs at the CMHI and in the Mazovian voivodeship.

**Figure S2** Distribution of the SARS-CoV-2 variants in Poland during the study period (data source: ECDC. Data on SARS-CoV-2 variants in the EU/EEA. https://www.ecdc.europa.eu/en/publications-data/data-virus-variants-covid-19-eueea)

1. von Elm, E. et al. The strengthening the reporting of observational studies in epidemiology (STROBE) statement: Guidelines for reporting observational studies. Int. J. Surg. 12, (2014). [↑](#footnote-ref-1)
2. European Centre for Disease Prevention and Control. *Interim analysis of COVID-19 vaccine effectiveness in healthcare workers, an ECDC multi-country study, May 2021- July 2022. ECDC: Stockholm;2022*. (2022). [↑](#footnote-ref-2)
