# Supplementary figures and images for: Incidence of SARS-CoV-2 infection among healthcare workers before and after COVID-19 vaccination in a tertiary paediatric hospital in Warsaw: A retrospective cohort study
Source: PLoS One. 2024 May 23;19(5):e0301612. doi: 10.1371/journal.pone.0301612 (PMC11115228; doi:10.1371/journal.pone.0301612)

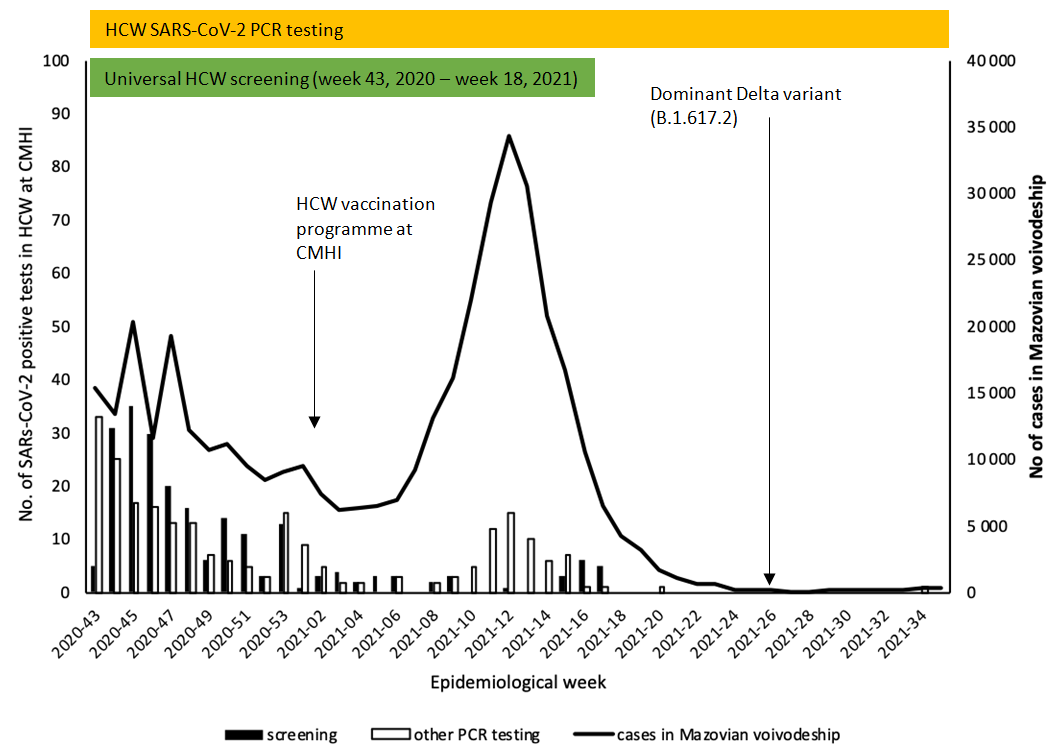

Supplement: S1 Fig — (TIF) [file pone.0301612.s002.tif]

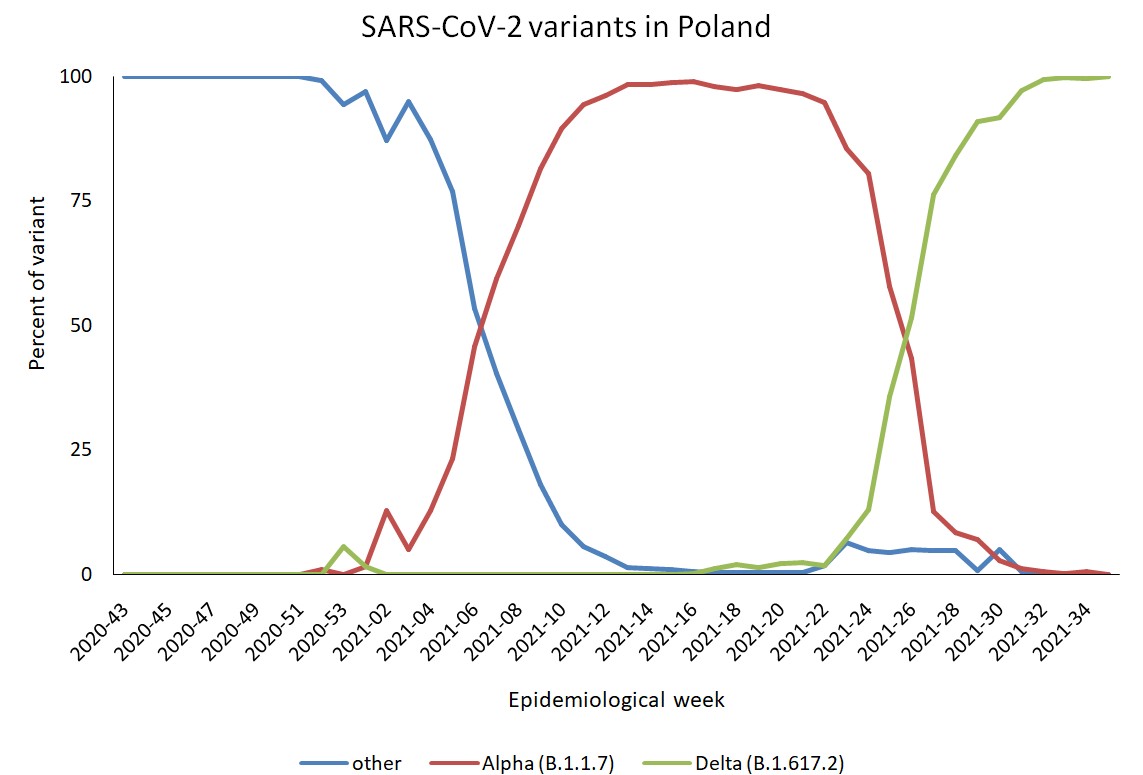

Supplement: S2 Fig — Data on SARS-CoV-2 variants in the EU/EEA. https://www.ecdc.europa.eu/en/publications-data/data-virus-variants-covid-19-eueea). (TIF) [file pone.0301612.s003.tif]
