# Supplementary material for: Incidence of SARS-CoV-2 infection among healthcare workers before and after COVID-19 vaccination in a tertiary paediatric hospital in Warsaw: A retrospective cohort study
Source: PLoS One. 2024 May 23;19(5):e0301612. doi: 10.1371/journal.pone.0301612 (PMC11115228; doi:10.1371/journal.pone.0301612)
Supplement: S1 Table — (DOCX) [file pone.0301612.s004.docx]

**S1 Table. Characteristics of the HCWs by SARS-CoV-2 infection status including 86 previously infected HCWs (sensitivity analysis, n = 2233).**

| **Characteristics** | **Infected** | **Uninfected** | **p-value** |
| --- | --- | --- | --- |
| Total, n (%): | 443 (19.8) | 1790 (70.2) |  |
| Age, median (IQR), years: | 47.0 (37.8–54.7) | 47.3 (36.6–55.8) | 0.651 |
| Female gender, n (%): | 385 (20) | 1 495 (80.0) | 0.080 |
| Professional category, n (%): |  |  | < 0.001 |
| nurse | 176 (24.0) | 556 (76.0) |  |
| physician | 64 (13.0) | 413 (87.0) |  |
| other with direct patient contact | 62 (21.0) | 234 (79.0) |  |
| other without direct patient contact | 141 (19.0) | 587 (81.0) |  |
| Working in COVID-19 area, n (%): |  |  | 0.058 |
| yes | 45 (25.0) | 133 (75.0) |  |
| no | 398 (19.0) | 1657 (81.0) |  |
| Hospital department, n (%): |  |  | 0.569 |
| clinical | 325 (20.0) | 1289 (80.0) |  |
| non-clinical | 118(19.0) | 501 (81.0) |  |
| Wards, n (%): |  |  | 0.069 |
| medical | 187 (20.0) | 752 (80.0) |  |
| surgical | 41 (22.0) | 142 (78.0) |  |
| intensive care | 33 (27.0) | 89 (73.0) |  |
| auxiliary | 38 (15.0) | 208 (85.0) |  |
| ambulatory | 26 (21.0) | 98 (79.0) |  |
| laboratory | 28 (22.0) | 99 (78.0) |  |
| maintenance | 26 (27.0) | 71 (73.0) |  |
| administration | 55 (16.0) | 288 (84.0) |  |
| other | 9 (17.0) | 43 (83.0) |  |
| Symptoms, n (%): |  |  |  |
| yes | 190 (42.9) | - |  |
| no | 231 (52.1) | - |  |
| unknown | 22 (4.9) | - |  |

Abbreviation: IQR – interquartile range

The percentages were presented in rows to highlight the proportion of infected and uninfected HCWs for each level of the variables (not applicable to the symptoms category)
