# Supplementary material for: Incidence of SARS-CoV-2 infection among healthcare workers before and after COVID-19 vaccination in a tertiary paediatric hospital in Warsaw: A retrospective cohort study
Source: PLoS One. 2024 May 23;19(5):e0301612. doi: 10.1371/journal.pone.0301612 (PMC11115228; doi:10.1371/journal.pone.0301612)
Supplement: S2 Table — (DOCX) [file pone.0301612.s005.docx]

**S2 Table. Association of demographic and occupational characteristics of the HCWs with SARS-CoV-2 infection before vaccination (study phase 1).**

| **Characteristics** | **Total**  **n = 2147** | **Infected, n (%)**  **n = 357 (16.6)** | **OR (95% CI)** | **p-value** | **aOR (95% CI) ^a,b^** | **p-value** |
| --- | --- | --- | --- | --- | --- | --- |
| Gender: |  |  |  |  |  |  |
| male | 341 | 46 (13.5) | ref |  |  |  |
| female | 1806 | 311 (17.2) | 1.33 (0.96–1.86) | 0.091 | 1.08 (0.72–1.62) | 0.703 |
| Median age (IQR), years: | 47.2 (36.6–55.6) | 46.6 (37.1–54.8) | 1.00 (0.99–1.01) | 0.532 |  |  |
| Professional category: |  |  |  |  |  |  |
| nurse | 695 | 139 (20.0) | 1.22 (0.93–1.60) | 0.145 | 1.80 (1.29–2.52) | 0.001 |
| physician | 459 | 46 (10.0) | 0.55 (0.38–0.78) | 0.001 | 0.45 (0.30–0.68) | < 0.001 |
| other with direct patient contact | 286 | 52 (18.2) | 1.09 (0.76–1.56) | 0.649 | 0.78 (0.51–1.20) | 0.260 |
| other without direct patient contact | 707 | 120 (17.0) | ref |  |  |  |
| Hospital department: |  |  |  |  |  |  |
| clinical | 1549 | 260 (16.8) | 1.04 (0.81–1.34) | 0.753 | - | - |
| non-clinical | 598 | 97 (16.2) | ref |  |  |  |
| Working in COVID-19 area: |  |  |  |  |  |  |
| yes | 165 | 32 (19.4) | 1.23 (0.82–1.84) | 0.321 | - | - |
| no | 1982 | 325 (16.4) | ref |  |  |  |
| Wards: |  |  |  |  |  |  |
| medical | 901 | 149 (16.5) | 1.17 (0.82–1.65) | 0.394 | - | - |
| surgical | 172 | 30 (17.4) | 1.24 (0.76–2.04) | 0.393 | - | - |
| intensive care | 111 | 22 (19.8) | 1.45 (0.83–2.53) | 0.188 | - | - |
| auxiliary | 242 | 34 (14.1) | 0.96 (0.60–1.54) | 0.868 | - | - |
| ambulatory | 123 | 25 (20.3) | 1.50 (0.88–2.56) | 0.137 | - | - |
| laboratory | 120 | 21 (17.5) | 1.25 (0.71–2.18) | 0.440 | - | - |
| maintenance | 90 | 19 (21.1) | 1.57 (0.87–2.84) | 0.132 | - | - |
| administration | 337 | 49 (14.5) | Ref |  |  |  |
| other | 51 | 8 (15.7) | 1.09 (0.49–2.47) | 0.829 | - | - |
| Median no. of PCR tests per person, n (IQR) | 4 (3–5) | 2 (1–3) | 0.37 (0.34–0.41) | < 0.001 | 0.35 (0.31–0.39) | < 0.001 |

Abbreviations: OR – odds ratio; aOR – adjusted odds ratio; CI – confidence interval; IQR – interquartile range; ref – reference category

^a^ adjustment for age, gender, and number of PCR tests per person

^b^ “-”, variable not included in the multivariate analysis model
