# Supplementary material for: Incidence of SARS-CoV-2 infection among healthcare workers before and after COVID-19 vaccination in a tertiary paediatric hospital in Warsaw: A retrospective cohort study
Source: PLoS One. 2024 May 23;19(5):e0301612. doi: 10.1371/journal.pone.0301612 (PMC11115228; doi:10.1371/journal.pone.0301612)
Supplement: S3 Table — (DOCX) [file pone.0301612.s006.docx]

**S3 Table. Characteristics of vaccinated and unvaccinated HCWs (n = 1461).**

| **Characteristics** | **Vaccinated ^a^** | **Unvaccinated ^b^** | **p-value** |
| --- | --- | --- | --- |
| Total, n (%) | 1345 | 116 |  |
| Age, median (IQR), years: | 48.6 (38.2–57.0) | 46.8 (35.8–54.3) | 0.001 |
| Female gender, n (%) | 1128 (91.8) | 101 (8.2) | 0.365 |
| Professional category, n (%): |  |  | < 0.001 |
| nurse | 407 (92.5) | 33 (7.5) |  |
| physician | 327 (97.6) | 8 (2.4) |  |
| other with direct patient contact | 163(89.1) | 20 (10.9) |  |
| other without direct patient contact | 448 (89.1) | 55 (10.9) |  |
| Working in COVID-19 area, n (%): |  |  | 0.804 |
| yes | 96 (91.4) | 9 (8.6) |  |
| no | 1249 (92.1) | 107 (7.9) |  |
| Hospital department, n (%): |  |  | 0.006 |
| clinical | 963 (93.3) | 69(6.7) |  |
| non-clinical | 382 (89.0) | 47(11.0) |  |
| Wards, n (%): |  |  | 0.224 |
| medical | 569 (93.0) | 43 (7.0) |  |
| surgical | 113 (96.6) | 4 (3.4) |  |
| intensive care | 62 (92.5) | 5 (7.5) |  |
| auxiliary | 147 (93.6) | 10 (6.4) |  |
| ambulatory | 73 (91.3) | 7 (8.7) |  |
| laboratory | 86 (90.5) | 9 (9.5) |  |
| maintenance | 53 (86.9) | 8 (13.1) |  |
| administration | 213 (89.1) | 26 (10.9) |  |
| other | 30 (88.2) | 4 (11.8) |  |

Abbreviation: OR – odds ratio.

^a^ received at least one dose of the BNT162b2 vaccine between January 4, 2021, and February 28, 2021

^b^ did not receive any dose of vaccine between January 4, 2021, and February 28, 2021

***NOTE*:** the definition of vaccination status was different for the incidence study in which the status at the time of PCR testing was considered
