# Supplementary material for: Incidence of SARS-CoV-2 infection among healthcare workers before and after COVID-19 vaccination in a tertiary paediatric hospital in Warsaw: A retrospective cohort study
Source: PLoS One. 2024 May 23;19(5):e0301612. doi: 10.1371/journal.pone.0301612 (PMC11115228; doi:10.1371/journal.pone.0301612)
Supplement: S4 Table — (DOCX) [file pone.0301612.s007.docx]

**S4 Table. Characteristics of the HCWs stratified by SARS-CoV-2 infection status during study phase 2 (postvaccination; n = 1461).**

| **Characteristics** | **Infected** | **Uninfected** | **Total** | **p-value** |
| --- | --- | --- | --- | --- |
| Total, n (%) | 93 (6.4) | 1368 (93.6) | 1461 |  |
| Age, median (IQR), years: | 45.1 (35.9–53.3) | 48.6 (38.1–56.9) | 48.3 (37.8–56.6) | 0.062 |
| Female gender, n (%): | 81 (6.6) | 1 148 (93.4) | 1 229 | 0.417 |
| Professional category, n (%): |  |  |  | 0.544 |
| nurse | 30 (6.8) | 410 (93.2) | 440 |  |
| physician | 16 (4.8) | 319 (95.2) | 335 |  |
| other with direct patient contact | 11 (6.0) | 172 (94.0) | 183 |  |
| other without direct patient contact | 36 (7.2) | 467 (92.8) | 503 |  |
| Hospital department, n (%): |  |  |  | 0.690 |
| clinical | 64 (6.2) | 968 (93.8) | 1032 |  |
| non-clinical | 29 (6.8) | 400 (93.2) | 429 |  |
| Working in COVID-19 area, n (%): |  |  |  | 0.485 |
| yes | 5 (4.8) | 100 (95.2) | 105 |  |
| no | 88 (6.5) | 1268 (93.5) | 1356 |  |
| Wards, n (%): |  |  |  | 0.771 |
| medical | 38 (6.2) | 573 (93.8) | 611 |  |
| surgical | 6 (5.1) | 111 (94.9) | 117 |  |
| intensive care | 4 (6.0) | 63 (94.0) | 67 |  |
| auxiliary | 9 (5.7) | 148 (94.3) | 157 |  |
| ambulatory | 7 (8.8) | 73 (91.2) | 80 |  |
| laboratory | 4 (4.2) | 91 (95.8) | 95 |  |
| maintenance | 2 (3.3) | 59 (96.7) | 61 |  |
| administration | 20 (8.4) | 219 (91.6) | 239 |  |
| other | 3 (8.8) | 31 (91.2) | 34 |  |
| Vaccination status, n (%): |  |  |  | < 0.001 |
| unvaccinated | 50 (39.7) | 76 (60.3) | 126 |  |
| partially vaccinated | 6 (12.2) | 43 (87.8) | 49 |  |
| fully vaccinated | 37 (2.9) | 1 249 (97.1) | 1286 |  |
| Symptoms at positive PCR test result, n (%): |  |  |  |  |
| total | 69 (75.0) | - | 69 |  |
| unvaccinated | 38 (76.0) | - | 38 | 0.864 |
| partially vaccinated | 4 (66.7) | - | 4 |  |
| fully vaccinated | 27 (73.0) | - | 27 |  |
| Median no. of PCR tests per person, n (IQR) | 2 (1–3) | 2 (2–3) | 2 (2–3) | 0.002 |

Abbreviation: OR – odds ratio
