# Supplementary material for: Incidence of SARS-CoV-2 infection among healthcare workers before and after COVID-19 vaccination in a tertiary paediatric hospital in Warsaw: A retrospective cohort study
Source: PLoS One. 2024 May 23;19(5):e0301612. doi: 10.1371/journal.pone.0301612 (PMC11115228; doi:10.1371/journal.pone.0301612)
Supplement: S5 Table — (DOCX) [file pone.0301612.s008.docx]

**S5 Table. Association of vaccination status with primary laboratory confirmed SARS-CoV-2 infection, after inclusion of additional category of HCWs with missing vaccination status.**

| **Parameter** | **Total,**  **n** | **Infected,**  **n (%)** | **Uninfected,**  **n (%)** | **p-value** |
| --- | --- | --- | --- | --- |
| Vaccination status: |  |  |  | < 0.001 |
| fully vaccinated | 1286 | 37 (2.9) | 1249 (97.1) |  |
| partially vaccinated | 49 | 6 (12.2) | 43 (87.8) |  |
| unvaccinated | 126 | 50 (39.7) | 76 (60.3) |  |
| unknown | 192 | 0 (0) | 192 (100) |  |
