# Supplementary material for: Incidence of SARS-CoV-2 infection among healthcare workers before and after COVID-19 vaccination in a tertiary paediatric hospital in Warsaw: A retrospective cohort study
Source: PLoS One. 2024 May 23;19(5):e0301612. doi: 10.1371/journal.pone.0301612 (PMC11115228; doi:10.1371/journal.pone.0301612)
Supplement: S6 Table — (DOCX) [file pone.0301612.s009.docx]

**S6 Table. Association of demographic and occupational characteristics of HCWs with SARS-CoV-2 infection after vaccination (study phase 2).**

| **Characteristics** | **Total, n = 1461** | **Infected, n = 93** | **OR (95% CI)** | **P-value** | **aOR (95% CI)^a, b^** | **p-value** |
| --- | --- | --- | --- | --- | --- | --- |
| Median age (IQR), years | 48.3 (37.8–56.6) | 45.1 (35.9–53.3) | 0.98 (0.96–1.00) | 0.016 | 0.99 (0.97–1.01) | 0.201 |
| Gender, n (%): |  |  |  |  |  |  |
| male | 231 | 12 (5.2) | ref |  |  |  |
| female | 1229 | 81 (6.6) | 1.29 (0.69–2.41) | 0.418 | 1.15 (0.58–2.28) | 0.693 |
| Professional category, n (%): |  |  |  |  |  |  |
| nurse | 440 | 30 (6.8) | 0.95 (0.57–1.57) | 0.839 | - | - |
| physician | 335 | 16 (4.8) | 0.65 (0.36–1.19) | 0.164 | - | - |
| other with direct patient contact | 183 | 11 (6.0) | 0.83 (0.41–1.67) | 0.580 | - | - |
| other without direct patient contact | 503 | 36 (7.2) | ref |  |  |  |
| Hospital department, n (%): |  |  |  |  |  |  |
| clinical | 1032 | 64 (6.2) | 0.91 (0.58–1.44) | 0.691 | - | - |
| non-clinical | 429 | 29 (6.8) | ref |  |  |  |
| Working in COVID-19 area, n (%): |  |  |  |  |  |  |
| yes | 105 | 5 (4.8) | 0.72 (0.29–1.82) | 0.487 | - | - |
| no | 1356 | 88 (6.5) | ref |  |  |  |
| Wards, n (%): |  |  |  |  | - | - |
| medical | 611 | 38 (6.2) | 0.73 (0.41–1.28) | 0.266 | - | - |
| surgical | 117 | 6 (5.1) | 0.59 (0.23–1.52) | 0.274 | - | - |
| intensive care | 67 | 4 (6.0) | 0.70 (0.23–2.11) | 0.521 | - | - |
| auxiliary | 157 | 9 (5.7) | 0.67 (0.30–1.50) | 0.327 | - | - |
| ambulatory | 80 | 7 (8.8) | 1.05 (0.43–2.58) | 0.915 | - | - |
| laboratory | 95 | 4 (4.2) | 0.48 (0.16–1.45) | 0.193 | - | - |
| maintenance | 61 | 2 (3.3) | 0.37 (0.08–1.63) | 0.190 | - | - |
| administration | 239 | 20 (8.4) | ref |  |  |  |
| other | 34 | 3 (8.8) | 1.06 (0.30–3.78) | 0.929 | - | - |
| Vaccination status, n (%): |  |  |  |  |  |  |
| unvaccinated | 126 | 50 (39.7) | ref |  | ref |  |
| partially vaccinated | 49 | 6 (12.2) | 0.21 (0.08–0.54) | 0.001 | 0.21 (0.08–0.54) | 0.001 |
| fully vaccinated | 1286 | 37 (2.9) | 0.05 (0.03–0.07) | < 0.001 | 0.05 (0.03–0.09) | < 0.001 |
| Median no. of PCR tests per person, n (IQR) | 2 (2–3) | 2 (1–3) | 0.48 (0.37–0.62) | < 0.001 | 0.93 (0.70–1.24) | 0.628 |

Abbreviations: OR – odds ratio; aOR – adjusted odds ratio; CI – confidence interval; ref – reference category

^a^ adjustment for age, gender, and number of PCR tests per person

^b^ “-”, variable not included in the multivariate analysis model
